# Folding or holding?—Hsp70 and Hsp90 chaperoning of misfolded proteins in neurodegenerative disease

**DOI:** 10.1016/j.jbc.2022.101905

**Published:** 2022-04-06

**Authors:** Benjamin S. Rutledge, Wing-Yiu Choy, Martin L. Duennwald

**Affiliations:** 1 Department of Biochemistry, Western University, London, Ontario, Canada; 2 Department of Anatomy and Cell Biology, Western University, London, Ontario, Canada

**Keywords:** tau, alpha-synuclein, TDP-43, Hsp90, molecular chaperones, intrinsically disordered proteins, AD, Alzheimer’s disease, ALS, amyotrophic lateral sclerosis, Cdc37, cell division cycle protein 37, CTD, C-terminal domain, GR, glucocorticoid receptor, Hsp70, heat shock protein 70 kDa, Hsp90, heat shock protein 90 kDa, Hsps, heat shock proteins, IDP, intrinsically disordered protein, LBD, ligand-binding domain, LBs, lewy bodies, MD, middle domain, NAC, non-Aβ component, NB, nuclear body, NBD, nucleotide-binding domain, NMR, nuclear magnetic resonance, NR, NRLLLTG peptide, NTD, N-terminal domain, PD, Parkinson’s disease, RRM, RNA recognition motif, SBD, substrate-binding domain, TDP-43, Tar DNA-binding protein 43

## Abstract

The toxic accumulation of misfolded proteins as inclusions, fibrils, or aggregates is a hallmark of many neurodegenerative diseases. However, how molecular chaperones, such as heat shock protein 70 kDa (Hsp70) and heat shock protein 90 kDa (Hsp90), defend cells against the accumulation of misfolded proteins remains unclear. The ATP-dependent foldase function of both Hsp70 and Hsp90 actively transitions misfolded proteins back to their native conformation. By contrast, the ATP-independent holdase function of Hsp70 and Hsp90 prevents the accumulation of misfolded proteins. Foldase and holdase functions can protect against the toxicity associated with protein misfolding, yet we are only beginning to understand the mechanisms through which they modulate neurodegeneration. This review compares recent structural findings regarding the binding of Hsp90 to misfolded and intrinsically disordered proteins, such as tau, α-synuclein, and Tar DNA-binding protein 43. We propose that Hsp90 and Hsp70 interact with these proteins through an extended and dynamic interface that spans the surface of multiple domains of the chaperone proteins. This contrasts with many other Hsp90–client protein interactions for which only a single bound conformation of Hsp90 is proposed. The dynamic nature of these multidomain interactions allows for polymorphic binding of multiple conformations to vast regions of Hsp90. The holdase functions of Hsp70 and Hsp90 may thus allow neuronal cells to modulate misfolded proteins more efficiently by reducing the long-term ATP running costs of the chaperone budget. However, it remains unclear whether holdase functions protect cells by preventing aggregate formation or can increase neurotoxicity by inadvertently stabilizing deleterious oligomers.

The heat shock proteins (Hsps) are a family of molecular chaperones that ensure the correct folding of proteins under physiological conditions and refold misfolded polypeptides during stress periods (1). Hsps and proteases make up a large component of the cellular protein quality control system, which refolds or degrades misfolded proteins (2). We define misfolded proteins as proteins that attain a nonnative, often nonfunctional and cytotoxic conformation. When the generation of misfolded proteins exceeds the capacity of the cellular protein quality-control system, misfolded proteins accumulate, which can be toxic to cells. Many disease-associated proteins form amyloid fibrils that are characterized by highly ordered cross-β-sheet structures (3). These fibrils from aggregates that are a pathological hallmark of many neurodegenerative diseases, including Alzheimer’s disease (AD), amyotrophic lateral sclerosis (ALS), and Parkinson’s disease (PD) (4).

In many cases, intrinsically disordered proteins (IDPs) are responsible for the formation of these protein inclusions, aggregates, and fibrils, specifically tau, α-synuclein, and Tar DNA-binding protein 43 (TDP-43), which are found in neuropathological inclusions in neurons of AD, PD, and ALS patients, respectively (5). We thus define misfolded IDPs as those that accumulate in inclusions, aggregates, or fibrils and are often nonfunctional and cytotoxic. Unlike structured proteins, IDPs lack a well-defined native structure and sample an ensemble of conformations (6, 7, 8, 9). These IDPs are susceptible to aggregation when they fail to adopt their functional conformations (10), leading to the formation of polymorphic oligomers that can seed aggregation (11). Two members of the Hsp family, heat shock protein 90 kDa (Hsp90) and heat shock protein 70 kDa (Hsp70), maintain the solubility of tau, α-synuclein, and TDP-43, indicating their role in modulating potentially toxic protein accumulation.

Numerous review articles have addressed the potential roles of molecular chaperones in neurodegenerative diseases (2, 4, 5, 12). However, few biochemical mechanisms have been suggested to describe how Hsp90 and Hsp70 prevent the toxicity associated with protein misfolding or potentially even exacerbate their detrimental effects. This review summarizes recent structural and biochemical findings regarding the interaction between Hsp90 and Hsp70 with tau, α-synuclein, and TDP-43. The pathology of proteinopathies caused by these IDPs share numerous commonalities and represent a large range of diseases including tauopathies (13), synucleinopathathies (14), ALS, and frontotemporal lobar degeneration (15). We also suggest a model by which Hsp90 and Hsp70 recognize and bind misfolded IDPs, modulate their aggregation, or potentially contribute to disease progression.

## Foldases and holdases

Molecular chaperone function can be categorized into two distinct types: foldase, which requires ATP hydrolysis, and holdase, which is ATP independent. The energy-dependent foldase pathway assists the transition of nonnative client conformations to their native conformations. By contrast, the ATP-independent holdase mechanisms capture free unfolded proteins and maintains them in soluble forms. During slow growth conditions or slow or no cell division, a higher cellular concentration of holdases is suggested to be beneficial. Over longer time spans, the energy cost requirements of foldases can deplete the ATP budget, making holdases an energy-efficient pathway for controlling unfolded proteins (16). The energy-efficient holdase pathway is particularly important for differentiated neurons, which typically do not divide. Since the accumulation of misfolded proteins in nondividing neurons is not redistributed between mother and daughter cells as in rapidly dividing cells, neurons depend on molecular chaperones to manage misfolded protein levels throughout their lifespan (12). The only members of the heat shock family of chaperones that are obligate holdases are the small Hsps, which do not possess ATPase function. Hsp90, Hsp70, and other molecular chaperones regulate misfolded protein accumulation through a combination of holdase, foldase, and disaggregase activities (17).

## Hsp90 foldase function

**Figure 1:**
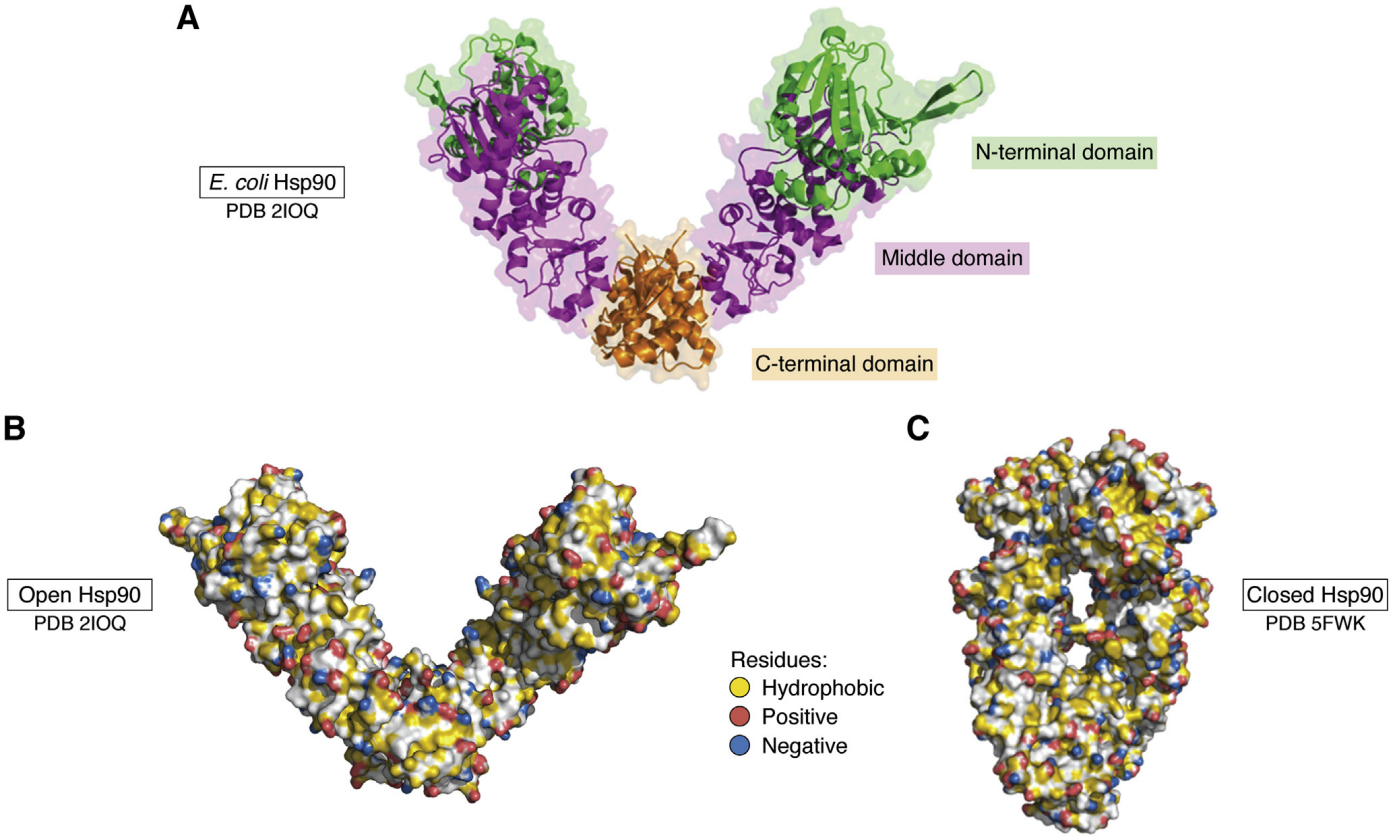
**Open and closed conformations of Hsp90.** Each monomeric unit of the Hsp90 dimer is comprised of three domains: the N-terminal domain (*green*), middle domain (*purple*), and C-terminal domain (*orange*) (*A*) (PDB: 2IOQ; *E. coli* Hsp90). The surface of the open (*B*) (PDB: 2IOQ) and closed (*C*) (PDB: 5FWK; human Hsp90 when in complex with Cdc37 and Cdk4) conformations of Hsp90 contained scattered patches of hydrophobic residues and both positive and negative charges, labeled in *yellow*, *blue*, and *red*, respectively using a script by Hagemans *et al.* (75). Cdc37, cell division cycle protein 37; Hsp90, heat shock protein 90 kDa.

Hsp90 functions as a homodimer, and each monomer consists of three domains: the N-terminal ATP-binding domain (NTD), the client and co-chaperone–binding middle domain (MD), and the C-terminal domain (CTD), which facilitates dimerization (18) (Fig. 1*A*). Hsp90 adopts its open, V-shaped conformation when it is not bound to ATP (19) (Fig. 1*B*). ATP-binding facilitates a series of dynamic conformational rearrangements throughout all three Hsp90 domains, causing Hsp90 to adopt a closed conformation in which the NTDs dimerize and associate with the MDs (20) (Fig. 1*C*). *In vivo*, Hsp90 mutants that lack ATPase activity cannot substitute for the WT protein, showing that the ATPase activity is essential to regulate the interaction between Hsp90 and clients (21, 22). While binding of ATP drives Hsp90 to form a molecular clamp that binds to clients, ATP hydrolysis facilitates the opening of Hsp90 and client release (18). Early mutagenesis studies suggest that the MD engages in client binding (23, 24), while there is now evidence of client binding of the NTD and CTD (20).

In contrast to other molecular chaperones, the general structural features of Hsp90 clients are poorly characterized. A high-throughput study of Hsp90 client interactions *in vivo* by Taipale *et al.* identified almost 400 Hsp90 client proteins. This analysis suggests that Hsp90 distinguishes client proteins based on their intrinsic stability rather than specific structural features or motifs (25). Typically, Hsp90 acts downstream of Hsp70, binding partially folded and less hydrophobic intermediates (26). Even though some studies indicate a more promiscuous nature to Hsp90 client recognition, Hsp90 interacts with a relatively limited number of clients compared to other Hsps, such as Hsp70.

**Figure 2:**
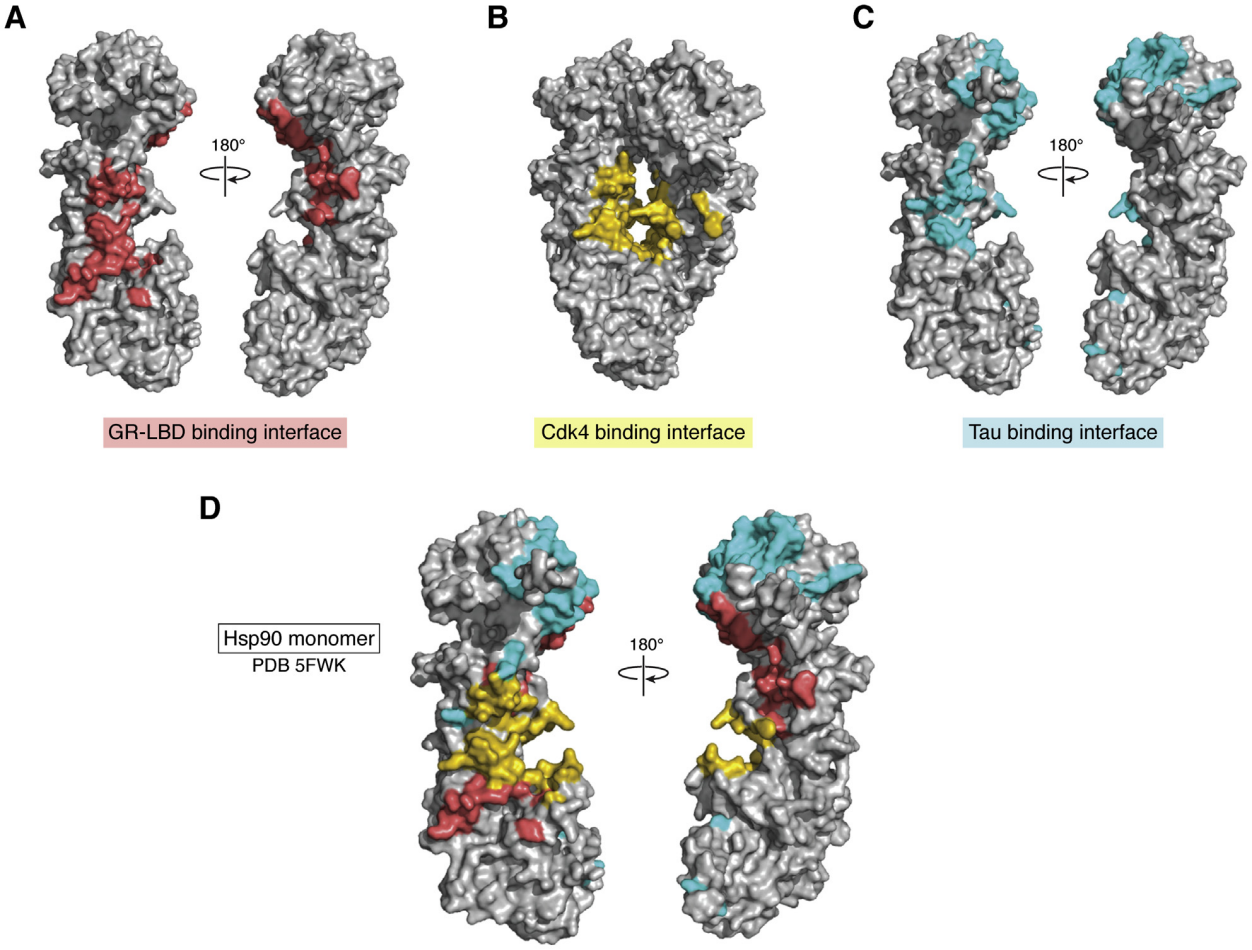
**The GR-LBD, Cdk4, and tau binding interfaces on Hsp90.***A*, the binding interface of GR-LBD on the Hsp90 monomer (19) is highlighted (*red*). *B*, the Cdk4 binding interface on Hsp90 is highlighted (*yellow*) on the Hsp90 dimer (29). *C*, the tau binding interface is highlighted (*cyan*) on the Hsp90 monomer (41). *D*, overlay of the binding interfaces of tau, GR-LBD, and Cdk4 on the Hsp90 monomer (PDB: 5FWK). GR, glucocorticoid receptor; Hsp90, heat shock protein 90 kDa; LBD, ligand-binding domain.

To date, only a few interactions between client proteins and Hsp90 have been structurally characterized. The glucocorticoid receptor (GR) has served as a model client for the Hsp90 foldase machinery. GR consists of three domains: the NTD, DNA-binding domain, and the ligand-binding domain (LBD). The GR-LBD has been used to model the interactions between Hsp90 and clients and the progression of the chaperone cycle. Even though the GR-LBD can bind weakly to the open conformation of Hsp90, the binding of ATP to the NTD triggers the transition of Hsp90 to the closed conformation and enhances the GR-LBD binding. The interaction between GR-LBD and Hsp90 involves amino acid residues mainly localized within the MD of Hsp90, which comprises the cleft between the Hsp90 subunits of the closed conformation (19) (Fig. 2*A*).

The structure of the complex of Hsp90, cell division cycle protein 37 (Cdc37), and the cyclin-dependent kinase Cdk4 also provides clues into Hsp90’s client recognition. The recruitment of Cdk4 to Hsp90 by Cdc37 is essential for kinase activation (27). The N-terminal and C-terminal lobes of Cdk4 bind to opposite ends of the Hsp90 monomer and are connected by a β-strand, which reaches through the binding cleft formed between two Hsp90 monomers where it forms hydrophobic interactions (28). The two lobes of Cdk4 associate with segments on the outer edges of the Hsp90 dimer spanning the region between the NTD and MD (Fig. 2*B*). The larger lobe of Cdk4 interacts with a hydrophobic patch in the MD of one Hsp90 monomer, while the other Cdk4 lobe interacts with segments of the NTD on its associated Hsp90 monomer (27, 28).

In summary, the canonical foldase function of Hsp90 is mediated by the ATP-dependent conformational exchange between the open and closed conformations (20). Foldase clients interact with a binding interface centered on the MD of Hsp90 (19, 27), binding to Hsp90 in the open conformation and being trapped in the cleft between the Hsp90 monomers in the closed conformation (19, 20).

## Hsp70 foldase function

**Figure 3:**
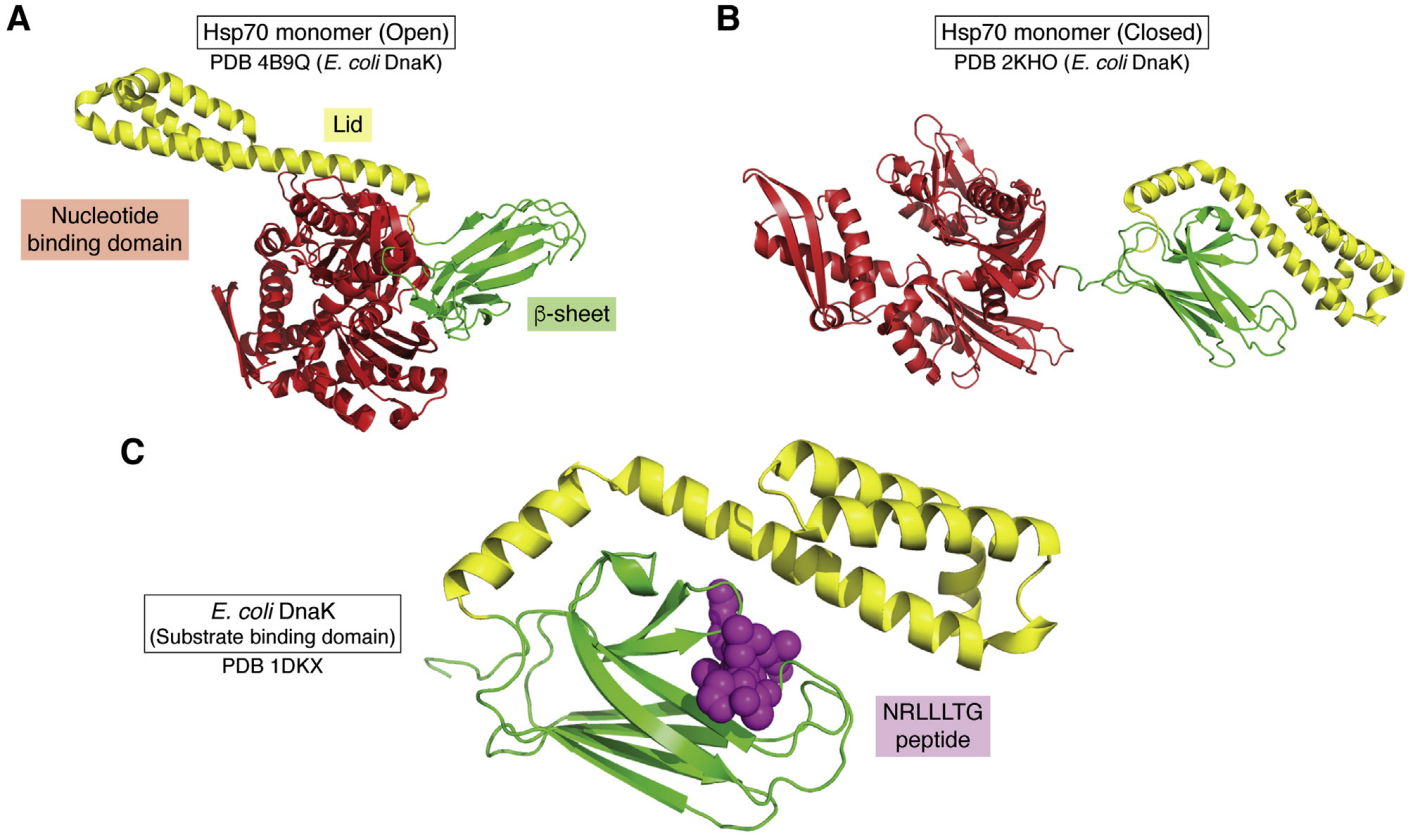
**The canonical folding mechanism of Hsp70.** Hsp70, represented using solved structures of *E. coli* Hsp70 homolog DnaK (31), is comprised of an NBD (*red*) and SBD, which consists of a lid (*yellow*) and beta-sheet (*green*) domain, that rearrange during the transition from the open conformation (*A*) (PDB: 4B9Q) and closed conformation (*B*) (PDB: 2KHO) following ATP hydrolysis (31, 32, 33). The crystal structure of the SBD of DnaK bound to the NRLLLTG peptide (*purple*) (PDB: 1DKX) acts as a model for the canonical substrate binding of Hsp70 (37) (*C*). Hsp70, heat shock protein 70 kDa; SBD, substrate-binding domain.

Hsp70 consists of two domains, the N-terminal nucleotide-binding domain (NBD) and the C-terminal substrate-binding domain (SBD), which are connected by a flexible linker. The SBD can be further divided into the β-sheet subdomain, which comprises the substrate-binding pocket, and the α-helical subdomain, which forms the lid (29) (Fig. 3*A*). The majority of biochemical and structural Hsp70 studies utilize the *E. coli* Hsp70 homolog, DnaK. Hsp70 and DnaK share ∼50% sequence identity and are highly similar in structure and function (30).

Conformational changes during the ATPase cycle of Hsp70 are required for Hsp70 chaperone function (30). During the ATPase cycle, Hsp70 transitions between an ATP-bound open conformation, with low substrate affinity and fast substrate exchange rates, and an ADP-bound closed conformation, with a high substrate affinity and slow substrate exchange rates (31). ATP hydrolysis causes conformational changes in the SBD, whereby the lid closes over the substrate-binding pocket and traps clients (30) (Fig. 3*B*). Truncations that remove the lid from the SBD reduce the affinity of Hsp70 for clients by up to 7-fold (32, 33, 34). Upon release of ADP and rapid binding of ATP, the lid folds away from the substrate-binding pocket, returning Hsp90 to its open conformation and releasing the bound substrate (31).

The central hydrophobic pocket of Hsp70 majorly contributes to its affinity to client proteins. Although the client-binding pocket is overall hydrophobic, the surface of that cavity also contains negative and positively charged groups, which increase client-binding affinity. Accordingly, clients with the highest affinity for Hsp70 have a hydrophobic core that is flanked by basic residues (30). Structures of Hsp70 bound to the model NRLLLTG peptide (Fig. 3*C*) reveal that the highly conserved L403, F428, V438, I440, I474, and V476 in the hydrophobic pocket of Hsp70 engage in van der Waals interactions with the central leucine residue of the substrate (35).

Of note, Hsp70 constructs lacking the lid domain maintain many of the characteristics of *in vitro* Hsp70 activities, including protein refolding. Remarkably, the Hsp70 constructs lacking the lid domain still show high or low substrate affinities dependent on the nucleotide state of the NBD, suggesting that the rearrangement of the lid domain between the ADP and ATP is not a required feature of the allostatic state (36). These findings have been supported by similar observations for lid-lacking DnaK constructs (37). It may be that the lid domain plays a larger role in protein–protein and protein–lipid interactions of Hsp70 than in its substrate binding (36).

In summary, the canonical foldase mechanism of Hsp70 is dependent on the ATP-dependent conformational transition between the open and closed conformations (30). The central pocket of the SBD, which contains highly conserved hydrophobic residues, mediates the substrate recognition and binding of Hsp70 (35).

## Shared foldase characteristics

There are many common characteristics between the canonical foldase activities of Hsp90 and Hsp70. The binding and release of substrates are mediated by conformational changes between open and closed states in an ATP-dependent process (19, 20, 23, 24, 30, 31). Hsp90 binds foldase substrates primarily through a canonical binding interface centered on the M domain (19, 27). Hsp70 interacts with foldase substrates through a well-defined central hydrophobic pocket within the SBD (35). Although relatively few Hsp90 and Hsp70 client interactions have been structurally characterized, it can be hypothesized that Hsp90 and Hsp70 foldase interactions are largely mediated through these canonical binding sites.

## Holdase function

Unlike the previous examples of foldase clients, IDPs lack a well-defined native structure. This raises the question, what foldase activity would Hsp90 and Hsp70 have on a client protein that is natively unfolded. Examining the binding interactions of Hsp90 and Hsp70 with unstructured clients such as tau, α-synuclein and TDP-43 reveals a noncanonical, ATP-independent holdase mechanism that is largely separate from foldase mechanisms outlined above.

## Hsp90 interaction with tau

The aggregation of microtubule-associated protein tau in neurons is one of the pathological hallmarks of AD. Under physiological conditions and in the absence of binding partners, tau is intrinsically disordered (38). Recent studies show that the unstructured tau is a *bona fide* client of Hsp90 with low micromolar binding affinity (39). However, unlike many other Hsp90 clients, the ATP-binding of Hsp90 does not increase its binding affinity to tau. Nuclear magnetic resonance (NMR) spectroscopy shows that the association between tau and Hsp90 covers an extended surface that spans both the NTD and MD, thus extending far into the NTD and even involving segments on the surface of the lid of the nucleotide-binding pocket (Fig. 2, *C* and *D*) (39). This extended interaction surface remains accessible for both the closed and open conformations of Hsp90 (26).

The interaction between tau and Hsp90 provides important insights into how IDPs can act as Hsp90 clients. The fact that no single point mutations within tau significantly alter its affinity for Hsp90 suggests that the affinity of tau is mediated by a large number of contacts in the extended interface and not through interactions with specific binding clefts (39). The surface of Hsp90 contains many hydrophobic patches that only allow binding for larger or extended proteins that can form a large number of low-affinity contacts with Hsp90. It has been suggested that this requirement for numerous contacts may remove the need for Hsp90 to conceal its binding interface (39). In addition to hydrophobic patches, the client-binding surface contains many positive and negative charges (Fig. 1, *B* and *C*). The Hsp90-binding surface is close to neutral charge, allowing it to bind both positive and negatively charged clients (26).

The idea that the binding affinity of tau for Hsp90 relies on a large number of low-affinity contacts is further supported by the fact that tau binding is dynamic (40). This contrasts with other misfolded client proteins for which only a single conformation bound to Hsp90 is proposed. Tau binds Hsp90 in various conformations, forming a “fuzzy” complex (41), *i.e.*, the binding of tau to Hsp90 is multivalent and polymorphic, allowing a broad assembly of tau conformations in complex with Hsp90 (40).

**Figure 4:**
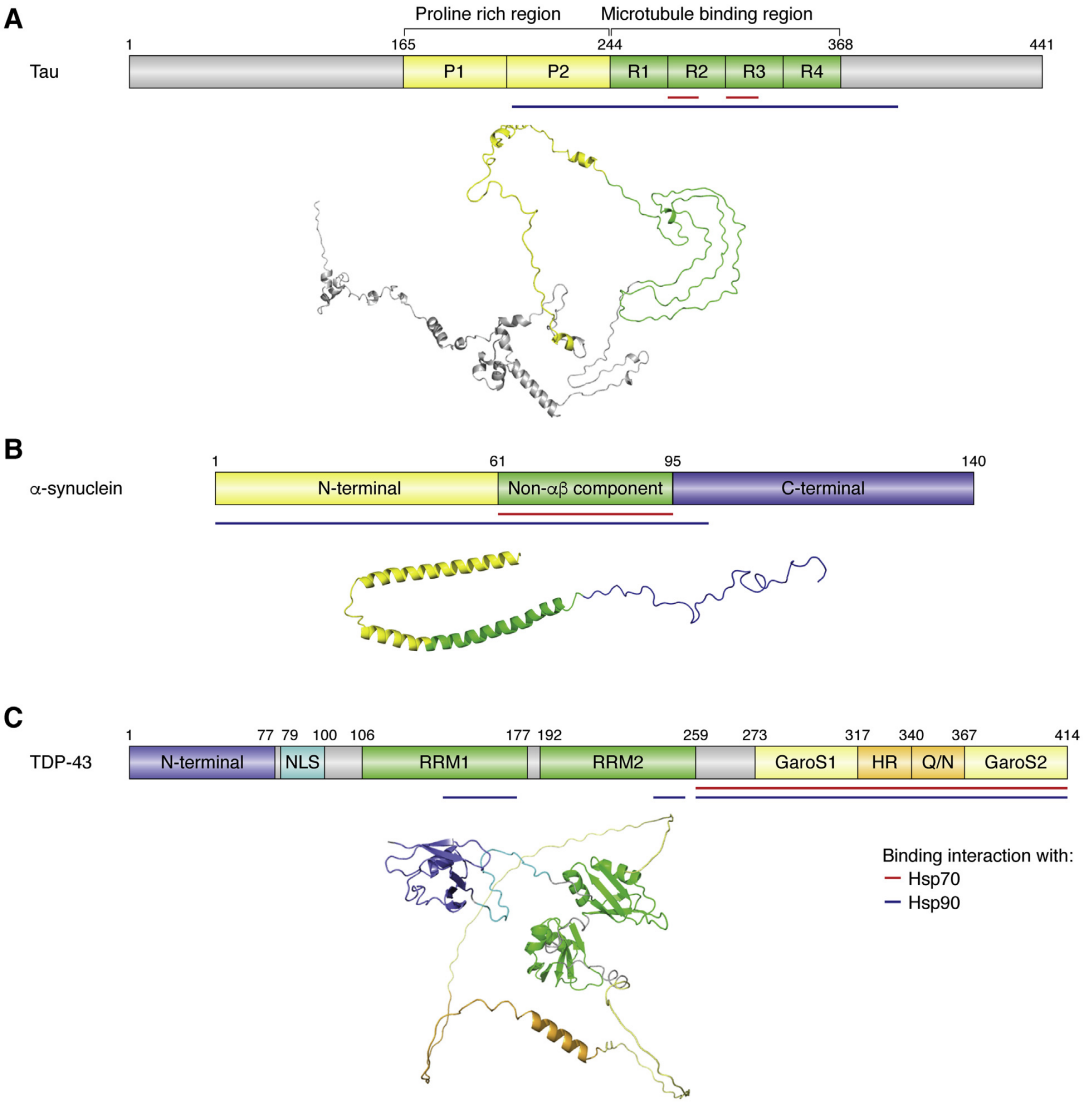
**Domain structure of tau, α-synuclein, and TDP-43.***A*, domain map of tau showing the relative sizes of the proline-rich region (PPR) comprised of P1 and P2 (*yellow*) and the microtubule binding region comprised of four tubulin binding domain repeats (R1–R4) (*green*). The location of the domains is shown using their respective colors on a tau structure generated using Robetta (76). *B*, domain map of α-synuclein showing the relative sizes of the N-terminal domain (NTD) (*yellow*), the non-Aβ component (NAC) core region (*green*), and the C-terminal domain (*blue*), with the respective domain locations shown on the α-synuclein structure (PDB: 1XQ8). *C*, domain map of TDP-43 showing the relative sizes of the NTD (*blue*), the nuclear localization sequence (NLS, *cyan*), the RNA recognition motifs (RRM1 and RRM2, *green*), the Gly-aromatic-Ser-rich regions (GaroS1 and GaroS2, light yellow), hydrophobic region (HR, *dark yellow*), and glutamine-arginine-rich region (Q/N, *dark yellow*), with the respective domain locations shown on the TDP-43 structure predicted by Alphafold (77). The regions of tau, α-synuclein, and TDP-43 believed to contribute to the binding interactions with Hsp90 (*purple*) and Hsp70 (*red*) are outlined on the respective domain maps of the proteins. Hsp70, heat shock protein 70 kDa; Hsp90, heat shock protein 90 kDa; TDP-43, Tar DNA-binding protein 43.

Tau does not require Hsp90 for folding. Rather, Hsp90 is responsible for supporting the interaction between tau and the microtubules or targeting tau for proteasomal degradation (39). Although tau is intrinsically disordered, the monomeric protein often does not attain a random coil conformation. Rather, long-range interactions mediate the folding over of terminal regions onto its central repeat domains (Fig. 4*A*), causing tau monomers to populate a “paper-clip” conformation. This “paper-clip” conformation protects against tau self-aggregation by shielding its aggregation-prone repeat domains (41). The binding of Hsp90 triggers the opening of the “paper-clip” conformation, which promotes the interaction with microtubules (39, 41). However, the opening of the paper-clip structure of tau within the tau/Hsp90 complex also exposes its central repeat domains, possibly promoting self-aggregation and the formation of deleterious oligomers. Specifically, binding of Hsp90 promotes the tau oligomerization *via* the repeat domains 3 and 4 (41). This same region is also involved in AD fibril formation (42).

Hsp90 also interacts with tau fibrils, altering their conformation away from the pathological state. Hsp90 inhibits fibril formation in a dose-dependent manner, preventing the formation of higher molecular weight tau species. In the presence of Hsp90, only smaller tau aggregates with decreased amyloid content are observed (43). Notably, in the presence of Hsp90, the abundance of many tau fibril-specific interactors across all functional clusters is reduced. Yet, interactions of tau monomers, but not fibrils, remain unaffected by Hsp90 or the formation of smaller aggregates (43). Together, this suggests that Hsp90 inhibits tau fibril formation by sequestering tau into smaller tau aggregates that maintain a more native conformation and possess the physiological interactome (43).

## Hsp90 interaction with α-synuclein

α-synuclein is another IDP associated with neurodegenerative diseases. The pathological aggregation of α-synuclein in the central nervous system is the hallmark of PD and related synucleinopathies (44). Although no structure has been solved of an α-synuclein–Hsp90 complex, much is known about the interactions between the two proteins. A recent study on the interactions of Hsp90 and five other molecular chaperones with α-synuclein by NMR revealed that the 12 amino acids in the N terminus and six residues around Tyr39 of α-synuclein are the common hydrophobic chaperone interaction motifs (45). The NMR results of another study suggested that Hsp90 interacts with multiple segments throughout the entire α-synuclein molecule, spanning the positively charged NTD (residue 1–60), the highly hydrophobic non-Aβ component (NAC) core region (residues 61–95), and a portion of the negatively charged CTD (69–105) (Fig. 4*B*). Similar to tau, α-synuclein does not bind Hsp90 in only a single conformation. Rather, Hsp90 binds and stabilizes an ensemble of partially folded α-synuclein conformations (46). The dynamic binding of α-synuclein and the large number of interacting residues suggests that, like tau, the affinity of α-synuclein for Hsp90 may originate from a large number of low-affinity interactions across an extended interface.

In cellular environments, α-synuclein exists in an equilibrium between its free, membrane-bound and chaperone-bound states. Based on the affinity of α-synuclein for Hsp90 and the cellular concentration of α-synuclein and chaperones, such as Hsp70 and Hsp90, it is estimated that up to 90% of the α-synuclein in neuronal synapses can be bound to chaperones (45). Hsp90 is the most prominent of all molecular chaperones to colocalize with α-synuclein in lewy bodies (LBs), as it is found in 95% of LBs (47). The interaction between α-synuclein and Hsp90 is not dependent on the closed Hsp90 conformation, since Hsp90 interacts with α-synuclein both in the absence and presence of ATP (46). This suggests that Hsp90 is always available for α-synuclein binding, similar to the tau/Hsp90 interaction. Notably, the regions of α-synuclein involved in binding Hsp90 are the same regions involved in lipid binding (NTD) (48) and amyloid formation (hydrophobic NAC core) (49). The binding of Hsp90 may also induce conformational changes of the N-terminal and middle domains of α-synuclein (46).

Falsone *et al.* showed that in the absence of ATP, Hsp90 binds α-synuclein and significantly suppresses fibril formation, further supporting that Hsp90 binds the same region responsible for amyloid formation. Unexpectedly, this study found that in the presence of ATP, Hsp90 strongly accelerated fibril formation. This result suggested a model by which Hsp90 regulates the α-synuclein fibril formation through two pathways: in the absence of ATP, Hsp90 stabilizes soluble oligomeric α-synuclein intermediates, whereas in the presence of ATP, Hsp90 accelerates their transition into fibrils (46).

Daturpalli *et al.* (50) confirmed that Hsp90 not only inhibits α-synuclein aggregate formation independent of its ATPase activity but also that truncations of each individual Hsp90 domain suppress α-synuclein aggregation. This further suggests that like tau, α-synuclein interacts with Hsp90 through an extended interface that spans all three Hsp90 domains. When α-synuclein aggregation is induced by agitation, in the absence of Hsp90, only 30% of the protein remains soluble after 7 days, whereas in the presence of Hsp90, up to 75% remains soluble. Accordingly, when SH-SY5Y cells are treated with α-synuclein from day 7 of aggregation in the presence of Hsp90, cell survival matched untreated cells, whereas in the absence of Hsp90, cell survival was reduced by 50% (50).

In contrast to Falsone *et al.*, Daturpalli *et al.* (50) found that Hsp90 reduced α-synuclein aggregation both in the absence and presence of ATP and posited that Mg^2+^ ion bound to ATP increases fibril formation. It is important to note that Daturpalli *et al.* used the pathological α-synuclein A53T variant while Falsone *et al.* tested WT α-synuclein. Regardless, both studies suggest that in cells, Hsp90 binds α-synuclein oligomers and stabilizes them in a nontoxic conformation, protecting them from further aggregation.

## Hsp90 interaction with TDP-43

TDP-43 is a partially disordered protein associated with ALS and other neurodegenerative diseases. The interaction between Hsp90 and TDP-43 has not been as extensively studied as tau and α-synuclein. Coimmunoprecipitation studies suggest that Hsp90 and TDP-43 associate in cells (51). It is estimated that as much as 30% of the full-length TDP-43 protein and that up to 66% of the C-terminal domain is disordered (52). The disordered C-terminal domain (residues 260–414) consists of multiple subdomains: the amyloidogenic core, broken down into the hydrophobic region (residues 318–340) and Q/N rich region (residues 341–367), and two GaroS regions (residues 273–317 and 368–414) (Fig. 4*C*). Although largely disordered, the C-terminal domain can form many well-ordered conformations that are able to self-associate (52). Based on the binding interfaces for tau and α-synuclein, it is tempting to speculate that the disordered C-terminal domain of TDP-43 binds Hsp90 in an extended conformation, forming numerous hydrophobic contacts.

Carlomagno *et al.* demonstrate that Hsp90 can significantly reduce the number and length of TDP-43 fibrils induced by CKII phosphorylation. Not only do these results suggest that Hsp90 protects against TDP-43 aggregation but also that the aggregation-prone phosphorylated TDP-43 binds to Hsp90 (53). Both RNA recognition motifs (RRM1 & RRM2) of TDP-43 are well-folded domains connected by a flexible linker. However, amyloidogenic cores in the RRM domains, *i.e.*, residues 166 to 173 in RRM1 and 246 to 255 in RRM2, contribute to misfolding and participate in TDP-43 aggregation. Residues 166 to 173 in RRM1 form a reasonably accessible loop (50% accessibility); however, residues 246 to 255 of RRM2 are buried in the folded state (27% accessibility) (52). The phosphorylation of TDP-43 and the ensuing conformational changes in these domains (52) may allow Hsp90 to interact with the exposed hydrophobic residues and protect TDP-43 from aggregation.

*In vivo* studies show that inhibiting Hsp90 ATPase activity or decreasing Hsp90 levels increases TDP-43 toxicity, suggesting that maintaining a balanced Hsp90 level is imperative for protecting against TDP-43 accumulation. On the other hand, overexpression of Hsp90 can facilitate aggregation by increasing TDP-43 levels (54). Furthermore, manipulation of Hsp co-chaperones, such as Cdc37, and stress-inducible phosphoprotein 1, which reduce Hsp90 ATPase activity, also affect TDP-43 activity. Overexpression of stress-inducible phosphoprotein 1 reduces TDP-43 aggregation and cytotoxicity (54). Alternatively, lowering Cdc37 levels destabilizes TDP-43, reducing its cytotoxicity (51). These results indicate that cells require a strict balance of Hsp90 and Hsp90 co-chaperone levels to clear misfolded TDP-43 rather than maintaining the stability of the toxic form of TDP-43 (51, 54).

## Hsp70 interaction with tau

The Hsp70 binding sites of tau localize to the repeat two and three domains (55). The segment KVQIINKK in the repeat two domain binds to the canonical Hsp70 binding site (Fig. 4*A*). Upon binding, the second isoleucine of the KVQIINKK motif becomes buried in the binding pocket of Hsp70 (36). This binding interaction mirrors that of the model NRLLLTG peptide (NR) with DnaK (37); however, there are structural differences between the two complexes. Moreover, the NRLLLTG peptide does not bind tightly to Hsp70, suggesting a discrepancy between the bacterial chaperone and its eukaryotic homolog regarding substrate recognition (36). The second Hsp70 binding sequence, VQIVYK in the repeat three domain (Fig. 4*A*) binds weakly (EC50 > 50 μM) to the SBD of Hsp70. Notably, the higher affinity KVQIINKK (KD ≈ 0.4 μM) sequence is not present in all isoforms of tau due to alternative splicing (36). This suggests that isoforms lacking the KVQIINKK sequence have a lower affinity for Hsp70 or contain alternative unidentified Hsp70 binding sequences (55).

Hippocampal sections from AD patients are immunopositive for either Hsp70 or aggregated tau, but rarely show both proteins together, suggesting that Hsp70 reduces the levels of insoluble tau (56). Recent studies have demonstrated that Hsp70 inhibits tau aggregation in a dose-dependent manner if added before inducing aggregation (57, 58). Furthermore, tau aggregates that form in the presence of Hsp70 are smaller, and the kinetics of tau fibril elongation is markedly reduced in the presence of Hsp70 (57). These inhibitory effects of Hsp70 on aggregation are not significantly altered by the presence of ATP. Although Hsp70 can reduce fibril formation and elongation, it does not effectively inhibit oligomer formation (58). The complex formed between Hsp70 and tau oligomers was found to be stable, in contrast to the transient complex formed with tau monomers. Hsp70 binding does not appear to differ between the oligomeric and fibril species, and the affinity of Hsp70 increases with aggregate size, whereas it is not significantly altered by structural differences, such as β-sheet content (57). The ability of substoichiometric concentration of Hsp70 to inhibit fibril formation suggests that Hsp70 sequesters tau in stable “off-pathway aggregates” (58). This is supported by the ability of Hsp70 to sequester tau oligomers and prevent them from perturbing lipid bilayers, thus reducing tau toxicity (57). This sequestration is an ATPase-independent process and is accomplished through the holdase function of Hsp70.

The complete Hsp70 disaggregation machinery, consisting of Hsp70, DNAJB1, and HSPA4, disassembles tau fibrils in an ATP-dependent process. The disaggregase activity is not specific to distinct tau variants and functions toward all tau isoforms, including pathological tau species extracted from AD patients. The tau species liberated from the fibrils by the Hsp70 disaggregation machinery are not only monomeric but also dimers and trimers. When introduced into a HEK293 cell model, these liberated monomers and small oligomers can form seeds (59). Furthermore, inhibiting the ATPase activity of Hsp70 dramatically reduces the cellular levels of tau, whereas activating it actually preserves it (60). Accordingly, this foldase-mediated disaggregation could be detrimental due to the seeding of additional aggregates *in vivo* (59).

## Hsp70 interaction with α-synuclein

The interaction between Hsp70 and α-synuclein was initially identified through the co-localization of Hsp70 with α-synuclein in LBs (61). Later studies have shown that Hsp70 inhibits α-synuclein fibril formation in a dose-dependent manner (49, 62, 63, 64). This suppression of α-synuclein fibrillation is nucleotide independent as the presence of ATP does not significantly alter the inhibitory effects (49). Furthermore, Hsp70 truncations that lack the NBD are sufficient to inhibit fibril formation, suggesting that the inhibitory effects of Hsp70 are not mediated by an ATPase-dependent folding by Hsp70 (64). It has been well documented that the SBD (residues 386–640) alone is sufficient to inhibit fibril formation (49, 62, 64), even showing more efficient inhibition than full-length Hsp70 (64). By contrast, truncations of the SBD that lack the C-terminal lid domain (residues 386–543) show reduced suppression of α-synuclein fibrillation. Overexpression of full-length Hsp70, as well as the SBD alone, can inhibit the toxicity of α-synuclein accumulation in cells (62), whereas SBD constructs lacking the lid domain show reduced protection against α-synuclein toxicity (64).

The holdase activity of Hsp70 on α-synuclein was assumed to be mediated by the canonical substrate binding site in the SBD. In the ATP-bound state, the Hsp70 NBD interacts with the two subdomains of the SBD and separates the lid domain from the substrate-binding cleft. In the ADP-bound state, the lid closes over the substrate-binding cleft, forming the canonical binding pocket (62). Hsp70 was suggested to interact with α-synuclein and other holdase clients through the canonical binding site in the SBD (62). However, recent evidence illustrates an alternative binding interaction. Tao *et al.* demonstrated that α-synuclein does not compete with NR, a model peptide substrate for DnaK, for binding the canonical binding site in SBD. In addition, the inhibition of α-synuclein oligomerization by Hsp70 is not affected by the NR binding (62). By using different truncated forms and mutational variants of Hsp70, Tao *et al.* pinpointed that α-synuclein interacts with Hsp70 through a hitherto unmapped noncanonical binding interface spanning the β-sheet and lid domain of the SBD. Further, the binding of α-synuclein to Hsp70 is not affected by the nucleotide state of Hsp70, suggesting that this noncanonical binding site is available regardless of the domain organization of the SBD (62).

Hsp70 does not modulate the structural properties or disaggregate mature α-synuclein fibrils. Rather, Hsp70 inhibits fibril formation by interacting with prefibril α-synuclein intermediates (63). Though α-synuclein is intrinsically disordered and may possess exposed hydrophobic regions that bind Hsp70, Hsp70 does not interact with native α-synuclein (65). Monomeric α-synuclein is protected from self-aggregating by forming transient interactions whereby the terminal domains fold over and protect the aggregate-prone NAC core region (66), which is similar to the “paper-clip” conformation of tau. Also, the charged C-terminal of α-synuclein forms long-range interactions with the hydrophobic core (67). Perturbation of this protective conformation likely leads to the exposure of the hydrophobic core, facilitating the formation of prefibrillar α-synuclein intermediates and exposing regions involved in forming the α-synuclein-Hsp70 complex (65). In agreement with this model, Hsp70 can effectively suppress the aggregation of α-synuclein truncated variants that lack all N- or C-terminal regions that are not critical for fibril formation, suggesting that Hsp70 interacts directly with the NAC region (49). The ability for Hsp70 to inhibit fibril formation of truncated variants rule out the possibility of a foldase mechanism whereby α-synuclein is restored to a native state, confirming that Hsp70 protects against fibrillation as a holdase. α-synuclein oligomers do not form the major species resulting from Hsp70-mediated inhibition, rather small soluble α-synuclein species, resembling monomeric α-synuclein, are highly abundant (49).

## Hsp70 interaction with TDP-43

An interaction between Hsp70 and TDP-43 has been expected ever since the discovery of Hsp70-positive inclusions in the spinal cord neurons of sporadic ALS patients (68). However, not until recently has a binding mechanism for Hsp70 and TDP-43 been proposed by which Hsp70 binds TDP-43 in the nucleus, promoting nuclear body (NB) assembly and maintaining its liquid-like state (69). Removal of the disordered CTD of TDP-43 disrupts the ability of Hsp70 to maintain the liquid-like state of NBs, suggesting that Hsp70 interacts with TDP-43 through its CTD (Fig. 4*C*). The hydrophobic region between residues 320 to 340 shows the strongest interaction with Hsp70. Surprisingly, the NBD of Hsp70, rather than the canonical substrate-binding pocket of the SBD, shows the highest affinity for the hydrophobic region of TDP-43 (Fig. 4*C*). Also, the addition of ATP only moderately reduces the inhibitory effects of Hsp70 on TDP-43 aggregation. Thus, ATP may compete with TDP-43 for binding to the NBD of Hsp70 (70).

Together, the recent research into the interaction between TDP-43 and Hsp70 suggests that similar to the interaction between Hsp70 and α-synuclein, TDP-43 binds Hsp70 through a noncanonical binding interface. Furthermore, like α-synuclein, TDP-43 interacts with Hsp70 through a conserved hydrophobic region in the intrinsically disordered CTD. It is tempting to speculate that Hsp70 protects TDP-43 from aggregation through an ATP-independent holdase pathway because ATP competes with TDP-43 for binding to the NBD of Hsp70 (70).

Knockdown of Hsp70 by siRNA can increase TDP-43 levels in neuronal cells due to a reduction of TDP-43 clearance (68). Conversely, overexpression of Hsp70 effectively suppresses cytoplasmic TDP-43 oligomer and aggregate formation (71). Overexpression of Hsp70 also prevents nuclear TDP-43 inclusions from transitioning into hyperphosphorylated pathological aggregates without reducing the total number of TDP-43 NBs, suggesting that Hsp70 maintains the nonpathological liquid-like TDP-43 state of NBs (70).

## Holding or folding

Both Hsp70 and Hsp90 utilize ATP-dependent foldase pathways and ATP-independent holdase pathways to protect cells from the accumulation of misfolded proteins, including tau, α-synuclein, and TDP-43. Protein fibrils and amyloids were long believed to be the major toxic protein species in neurodegenerative diseases (72). Accordingly, this view led to the conclusion that the ATP-dependent disaggregase activity of Hsps is central in protecting cells. However, it is now understood that smaller oligomeric species are more toxic than fibrils (73), providing compelling evidence for the importance of the holdase pathways of Hsps.

Although tau, α-synuclein, and TDP-43 are examples of IDPs that are prone to aggregation, it is important to note that aggregation is not a common characteristic of all IDPs. Unlike other IDPs, tau, α-synuclein, and TDP-43 contain conserved hydrophobic regions that, when exposed, are prone to self-association and thus aggregation. The first line of defense for preventing aggregation is adopting conformations that shield these hydrophobic regions. IDPs typically do not form random structures in solution or in cells, as illustrated by the “paper-clip” conformation and the shielding of the hydrophobic region of tau and α-synuclein (41). Although Hsp70 interacts strongly with prefibril intermediates of tau and α-synuclein, the chaperone only forms transient interactions with monomeric tau (57) and does not bind monomeric α-synuclein (63, 65). Together, this suggests that tau and α-synuclein form nonrandom monomeric conformations that protect against both aggregation and the interaction with Hsp70, as both are mediated through the same shielded hydrophobic regions.

When these IDPs misfold, they expose the hydrophobic regions that mediate self-aggregation (42, 46, 70). However, the very same hydrophobic regions are also involved in the interactions with Hsp90 and Hsp70 (39, 46, 70), suggesting that Hsp70 and Hsp90 compete to bind the misfolded protein and prevent its accumulation into oligomers. The interactions between IDPs and Hsp70/Hsp90 are mediated by interfaces that are distinct from the canonical client binding domains of Hsp90 and Hsp70. Both tau and α-synuclein bind Hsp90 through an extended interface spanning all three Hsp90 domains (26). This binding interface is available in the ATP bound, ADP bound, and nucleotide-free states of Hsp90 (26, 46), suggesting that Hsp90 binds IDPs in a passive process independent from its regular foldase activities. Similarly, both α-synuclein and TDP-43 interact with noncanonical interaction surfaces on Hsp70 (62, 70). α-synuclein interacts with a region spanning the β-sheet domain and lid domain of the SBD (62), while TDP-43 interacts with the NBD of Hsp70 (70). These noncanonical interfaces are available regardless of the SBD conformation and effect peptide binding to the SBD (62), further suggesting a holdase pathway distinct from the canonical foldase chaperone function.

This holdase pathway allows Hsp90 and Hsp70 to prevent the aggregation of misfolded proteins in an ATP-independent pathway by binding the misfolded proteins and preventing their addition to oligomeric species. Furthermore, it allows Hsp90 and Hsp70 to bind oligomeric species and abrogate their toxicity. However, it is important to note that the holdase pathway does not seem to refold IDPs generally. Rather, the interaction between Hsp90 and tau induces tau to adopt an extended conformation resembling the pathological conformation, in which the hydrophobic repeat domains are exposed (41). Similarly, when α-synuclein interacts with Hsp90, α-synuclein undergoes secondary structure rearrangements in its N-terminal and hydrophobic middle domain (46), suggesting that α-synuclein also adopts an open conformation to bind the extended interface on Hsp90. Furthermore, Hsp70 binds *via* the hydrophobic regions of tau, α-synuclein, and TDP-43 that are normally shielded in the native conformation, indicating that the proteins are not in their native conformation when bound to the molecular chaperones. Together, this suggests that Hsp90 and Hsp70 bind the misfolded proteins and shield their hydrophobic regions, while maintaining them in a nonnative conformation. If not properly degraded, this could cause Hsp90 and Hsp70 to stabilize and maintain a population of deleterious prefibrillar species.

In the majority of the *in vitro* aggregation assays, the ATP-dependent activities of Hsp90 and Hsp70 are investigated in the absence of co-chaperones. However, in the presence of the complete disaggregase machinery, Hsp70 can disassemble tau fibrils. Since Hsp70 colocalizes with α-synuclein and TDP-43 fibrils, it is likely that under physiologically conditions, with all co-chaperones present, Hsp70 can also contribute to the disassembly of fibrils. Though this disaggregase activity may protect against the negative effects of fibril accumulation, the monomeric and oligomeric tau species liberated by Hsp70 can seed toxic aggregation in cells (58). The ATP-dependent disaggregase activity, like the holdase activity, may thus stabilize and maintain the toxic conformation of the liberated species, potentially contributing to disease progression.

**Figure 5:**
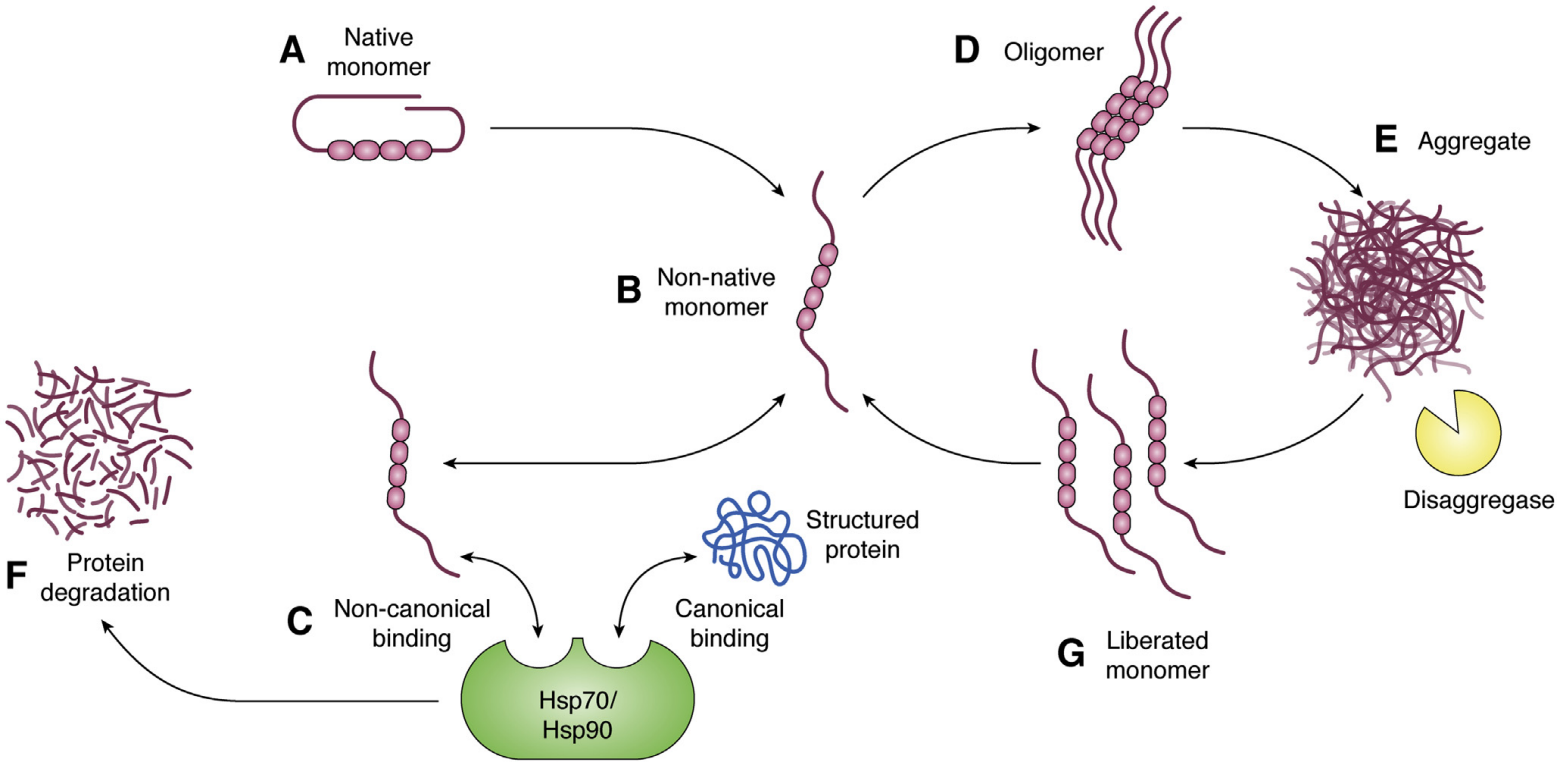
**The effect of the holdase function of Hsp90/70 on IDP aggregation.***A*, native conformation of the IDP monomer (tau shown) with hydrophobic regions shielded. *B*, nonnative monomeric conformation that exposes the hydrophobic regions. *C*, Hsp90/70 canonical binding interface, which interacts with structured foldase clients, and noncanonical binding interface, which interacts with the non-native IDP monomer through the holdase pathway. *D*, oligomeric species formed through the self-association of the exposed hydrophobic regions. *E*, aggregate (cellular inclusion, aggregate, or fibril formation) formation. *F*, degradation of the bound IDP. *G*, non-native monomeric IDPs liberated from the aggregates by the disaggregase machinery. Image created with BioRender.com. Hsp70, heat shock protein 70 kDa; Hsp90, heat shock protein 90 kDa; IDP, intrinsically disordered protein.

Though the ATP-dependent activities of Hsp90 and Hsp70 may have a more significant impact *in vivo*, *in vitro* studies suggest that it is largely the holdase activities of the chaperones that protect against the toxic accumulation of these misfolded proteins. Under physiological conditions, the first line of defense against misfolded IDPs may be the nonrandom native conformations, which protect the aggregate-prone hydrophobic regions. Once misfolding occurs, Hsp90 and Hsp70 bind the proteins through the interaction with extended binding interfaces, requiring a larger number of low-affinity interactions, mediated by the exposed hydrophobic groups within the misfolded conformation. Misfolded IDPs interact with noncanonical binding sites on Hsp90 and Hsp70 that seem to be constitutively available and independent of the canonical foldase activity of both chaperones. Hsp90 and Hsp70 protect the misfolded proteins from accumulating into toxic oligomers or fibrils; however, they can also maintain the misfolded protein in an extended monomeric conformation resembling the toxic prefibrillar species. The misfolded proteins can then be triaged for degradation. However, in pathological conditions, misfolded proteins accumulate and escape Hsp90 and Hsp70 to form oligomers and fibrils. Hsp70 and Hsp90 can bind oligomers and alleviate their toxicity, and eventually, the chaperones become overwhelmed. In the presence of co-chaperones, Hsp70 can bind and disassemble fibrils, although the species liberated by Hsp70 and other disaggregases can then facilitate the formation of new toxic oligomers, which exacerbate disease progression (Fig. 5).

## Conclusion

Although recent studies have helped elucidate the mechanisms by which Hsp70 and Hsp90 recognize and bind IDPs, there remain many important open questions. Experimental evidence supports a model in which chaperones, such as Hsp70 and Hsp90, bind tau, α-synuclein, and TDP-43 after they transition into a non-native conformation, thus exposing their aggregate-prone hydrophobic regions. However, according to an alternative model, Hsp70 and Hsp90 compete with the intramolecular interactions of the native tau, α-synuclein, and TDP-43 conformations and promote the opening of the proteins into a seeding-competent conformation. The interactions between IDPs and Hsps also suggest a nonspecific recognition system *via* elongated noncanonical binding regions that may mediate client recognition more often than previously expected. It is well-established that the human proteome contains many IDPs and many proteins with intrinsically disordered regions (74). Many of these IDPs may interact with chaperones, such as Hsps through similar noncanonical sites. Therefore, the noncanonical holdase functions of Hsp70 and Hsp90 could hint toward a larger client recognition mechanism that mediates protein interactions between IDPs and molecular chaperones beyond disease-associated misfolded proteins.

## Conflict of interest

The authors declare that they have no conflicts of interest with the contents of this article.
